# Editorial: Recent Research of Complex Structure Machining Technology for Hard-to-Machine Materials

**DOI:** 10.3390/ma17020340

**Published:** 2024-01-10

**Authors:** Shukai Fan, Xiaoyun Hu, Hansong Li

**Affiliations:** College of Mechanical & Electrical Engineering, Nanjing University of Aeronautics and Astronautics, Nanjing 210016, China

##  

With the development of machining technology, the application scenarios of national defense and military equipment, civil aviation vehicles, and reciprocating air and space vehicles are becoming more and more complicated [1]. High-tech fields have an increasing demand for materials with better thermal conductivity, specific stiffness, specific strength and other physical properties. With the continuous design innovations in the aerospace field, there is a growing demand for the machining of complex three-dimensional structures, especially for thin-walled parts with a high degree of integration, light weight and thin wall-thickness, such as for fuel storage tanks, aircraft fairings and other complex parts [2].

This Special Issue aims to explore advanced electrochemical machining methods for hard-to-machine materials. Meanwhile, in addition to electrochemical methods, there are also many processing methods for difficult-to-process materials, therefore, this editorial will center on several major categories of common processing methods for hard-to-machine materials.

Multi-axis CNC machining technology has the advantages of good machining flexibility, high precision and short preparation cycles, and is one of the most important methods for machining complex three-dimensional profile parts in the aerospace field. Researchers have studied the chip morphology and formation mechanisms, cutting forces and cutting temperatures [3] during the cutting process, and the machining efficiency can be improved via optimization of the structure of the cutting tool or adjusting the machining parameters during machining [4]. Covering the surface of the tool with a coating can also reduce tool wear and extend its life [5]. Zha investigated the transition from the alpha phase to beta phase during cutting Ti-6Al-4V, and suggested an optimal feed rate in TC4 machining [6]. Gao used supercritical carbon dioxide (sc CO_2_) as the cooling lubrication to mill Inconel 718 alloy, revealing that the use of sc CO_2_-based cooling and lubrication conditions significantly reduced the milling forces and milling temperatures and resulted in better machined surface quality [7].

Chemical milling technology using chemical corrosion achieves the selective, controlled dissolution of metal materials’ processing [8]. The surface of the workpiece does not need to be processed in relation to the protective coating that can resist corrosion solutions, after high temperature curing to form a protective film layer; furthermore, through scribing to determine the area to be machined [9]. However, the chemical milling process requires the use of strong corrosive solutions and will produce exhaust gases, which are not conducive to human health; consequently, these shortcomings limit the further development of the technology.

Mirror milling technology is a relatively new thin-wall machining technology developed in recent years, consisting of a flexible fixture and two synchronized-motion 5-coordinate machining machines [10]. The flexible fixture uses either a 3-coordinate flexible positioning vacuum chuck to adsorb and hold the part, or a peripheral flexible clamping frame to hold the part [11]. This technology has advantages over chemical milling in terms of machining accuracy, efficiency, environmental protection, energy saving, etc. It is also capable of machining some hard-to-machine composite parts, and its application in the field of aerospace manufacturing is gradually expanding [12,13]. Xiao presented a novel dual-robot mirror milling system that consisted of a machining hybrid robot, a supporting hybrid robot, and fixture, and proposed a collaborative machining for a triangular grid [14].

Electrical discharge machining (EDM) is a special machining technology that uses the high temperatures generated during the pulsed discharge between the conductive workpiece and the discharge tool to melt and etch away the metal material [15]. Electrical discharge machining can use a simple shape of the wire or rod electrode [16]; the tool cost is low and easy to prepare, having zero cutting force in the process of machining, and the technique is not subject to the physical and mechanical properties of the tool and the workpiece. In recent years, most of the research on this machining method has been carried out in the direction of reducing tool wear [17,18]. Anbalagan investigated the influence of the carbon fiber-reinforced polymer orientation and layering pattern when machined using the Wire-EDM machining process [19]. Pratap constructed an artificial neural network (ANN) model to help improve the performance of micro-blind hole machining, and the performance of EDM was improved through different process parameters and materials [20].

Through this Special Issue, we hope that more scholars will learn about the current research progress on the different machining methods of difficult-to-machine materials and will be attracted to participate in this Special Issue.
